# Factors associated with the mask-wearing behavior of university students in Japan: a cross-sectional study of the post-mask-mandate period

**DOI:** 10.3389/fpsyg.2025.1498560

**Published:** 2025-06-24

**Authors:** Yuko O. Hirano, Rin Iwashita, Yuina Muta, Minori Ishida

**Affiliations:** ^1^Nagasaki University Institute of Biomedical Sciences, Nagasaki, Japan; ^2^Nagasaki University School of Health Sciences, Nagasaki, Japan

**Keywords:** COVID-19, habit, health behavior, mask, Japan, university students

## Abstract

The coronavirus disease 2019 (COVID-19) pandemic has required people to adapt to a new lifestyle, which includes wearing masks. In Japan, mask-wearing mandates were prolonged for nearly 3 years, leading to the habitual use of masks by many people. This habit persisted even after the relaxation of the mandates. Excessive mask wearing, such as wearing masks in low-risk settings, can lead to dehydration, especially during the summer. However, studies on Japanese people’s mask-wearing behavior in the post-mask-mandate period are lacking. Therefore, the reasons that people excessively wear masks should be investigated. This study aimed to identify factors, including perceptions of mask wearing, that influence the frequency and habit of wearing masks in the post-mandate period. An online questionnaire was distributed to 471 university students in Japan between June 12 and 24, 2023. The results indicated that the frequency of mask wearing exhibited a dichotomous trend. According to the multiple regression analysis, the strongest predictor of both the frequency and habit of mask wearing was a sense of unease from not wearing a mask rather than anxiety related to COVID-19. This study provides the government with basic data to develop guidelines for the proper wearing of masks to prevent heatstroke.

## 1 Introduction

The coronavirus disease 2019 (COVID-19) pandemic, which was declared in early 2020, required people to not only combat the hazardous, life-threatening virus but also adapt to the “New Normal” conditions. The “New Normal” was a term coined to indicate how the pandemic completely transformed every aspect of human life globally, including professional identify, economic substance, work and family organization, and children’s education. This demanded a radical revision of traditional methods, practices, and skills used to manage them (Manuti et al., 2022). The World Health Organization (n.d.) have recommended wearing face masks to prevent viral transmission, and many governments followed these rules. In Japan, the “New Normal” was advocated by the government as a “new lifestyle,” one that should be adapted by individuals and businesses to remain healthy. The guidelines, published on May 4, 2020, included suggestions such as maintaining social distancing, wearing masks, washing hands thoroughly, and shifting to remote work and shift rotation (Saito, 2020).

The new lifestyle of mandatory mask wearing is challenging, even for medical professionals, who are accustomed to wearing masks while treating patients vulnerable to viral infections (Ekmektzoglou et al., 2021; Lewis et al., 2021; Mack and Fraser-Bell, 2021; Maru, 2021). Therefore, the impact on non-medical professionals who are not used to wearing masks is likely even greater due to the discomfort of wearing masks (Ribeiro et al., 2022). Previous studies on mask wearing in Western countries found that people had negative perceptions of mask wearing due to discomfort and vocal symptoms, such as difficulty in breathing and coordinating speech (Ribeiro et al., 2022). People in Western countries followed the mask-wearing recommendations of the authorities at the start of the COVID-19 pandemic (Goldberg et al., 2020); however, they were less likely to wear masks after the mandates were relaxed (Vest et al., 2022). People who had negative vaccine intentions were significantly less likely to wear masks (Latkin et al., 2021). A positive perception of the government was found to be associated with higher mask use in various countries (Wismans et al., 2022).

Long-term mask-wearing mandates may change people’s views of the concept of wearing masks by hindering the ability to recognize other people due to the mask covering one’s face (Freud et al., 2022). Cha et al. (2023) reported that mask wearing could shift from being a self-protection measure during the COVID-19 pandemic to a self-presentation tactic in the post-pandemic era. In other words, people wore masks primarily as a self-protective measure to prevent infection during the COVID-19 pandemic. However, in the post-pandemic era, mask wearing has evolved into a self-presentation tactic, used more broadly for general health and safety and as social or psychological comfort. Particularly in Japan, where many people wore masks before the COVID-19 pandemic (Chiyoma, 2019), the perceived attractiveness of people wearing masks increased after the onset of the pandemic (Kamatani et al., 2021). This suggests that Japanese people are more likely to have a positive perception of wearing masks than Western people (Crimon et al., 2022). Japanese people often wear masks, especially during pollen seasons, to protect themselves from pollen that may cause rhinitis, which is a national epidemic (Horii, 2014). During this time, in addition to protecting themselves from pollen, Japanese people present themselves as allergy sufferers who receive care from others. In other words, wearing a mask allows Japanese people to communicate their situation (Yoshikawa, 2017). The mask speaks about their positions and feelings without having to express them verbally.

Nakayachi et al. (2020) reported that Japanese people have several motivations to wear masks. They examined Japanese people for six possible psychological reasons, including three expectations of risk reduction: severity, protection, and prevention. Three driving psychological forces (norms, relief, and impulsion) for wearing masks during the COVID-19 pandemic were evaluated. They found that Japanese people’s mask wearing was motivated by socio-psychological incentives but not risk reduction. Furthermore, Sakakibara and Ozono (2021) reported that norms and impulsions significantly predicted mask wearing in Japanese people; however, severity, protection, and prevention did not. These studies suggest that Japanese people have unique behaviors regarding mask wearing driven more by social perceptions than by concerns about viral infections. Therefore, even after official announcements relaxing mask-wearing mandates, some Japanese people may feel uneasy or anxious or may perceive themselves as aloof to society if they do not wear masks. This may be especially true after a long period of mandatory mask wearing, during which they became accustomed to wearing masks and subsequently became afraid to remove them.

The perception of wearing masks can affect how people view mask use for self-presentation, particularly in Japan, which has a unique concept called *date-masuku* (Yoshikawa, 2017), literally translated as “fake mask.” *Date-masuku* is worn not for health reasons but for other purposes, such as following the social norms. This may promote the excessive use of masks, including in low-risk environments. Such behavior poses potential health risks, particularly during the summer months, as prolonged mask use can contribute to dehydration and other physiological burden (Ueno, 2021).

Therefore, the reasons that people excessively wear masks should be investigated. This study aimed to identify factors, including the perceptions of wearing masks, influencing the frequency and habit of wearing masks after the relaxation of the mask-wearing mandate on May 8, 2023, which was a response to the classification change of COVID-19 from the novel influenza and other disease category to the Class 5 category (Ministry of Health, Labor and Welfare, 2023). This study targets university students, a group whose behavior is often shaped by societal norms (Mashiko, 2010). Consequently, the findings may offer insights into broader societal trends in Japan.

## 2 Methods

### 2.1 Study participants

The target population for this study were students enrolled in a university in the western part of Japan.

### 2.2 Sampling procedure

This study conducted a questionnaire survey. For the sampling procedure, first, the authors listed the ID numbers of all students enrolled in the university. Each student in the university was assigned an ID number, which is also used to identify their email address. Second, random sampling was conducted to select 2,000 study participants from the list, aiming to select one-fourth of the students enrolled. Third, the link to an online questionnaire was distributed to the selected students via email, using the email address linked to their ID number.

### 2.3 Instruments

The online survey covered several topics. Frequency of wearing masks when going out was evaluated by asking “How often do you wear a mask when going out?” Responses were rated on a seven-point Likert scale (1 = never; 7 = always). The Self-Report Habit Index (SRHI) of mask wearing was used to measure the degree of development of mask-wearing habits. The 12-item SRHI scale was originally developed by Verplanken (2006) to measure the habit strength of health-related behaviors. The Japanese version of the scale was developed by Takami (2023). This study used the SRHI to measure the habit strength of wearing masks.

Gender, academic year, and academic course were used as control variables for mask-wearing behavior. In addition, we collected data on whether the respondents were required to wear masks when participating in practicums or part-time jobs at the time of the survey, frequency of mask-wearing before the onset of the COVID-19 pandemic due to pollen allergies or rhinitis, and whether they needed to wear masks due to medical conditions at the time of the survey.

COVID-19-related anxiety, a factor associated with mask-wearing behavior (Wang et al., 2020), was evaluated by asking “To what extent are you afraid of being affected by COVID-19 if you do not wear masks?” The sense of unease due to not wearing masks was measured by asking “How uneasy do you feel due to not wearing masks?” Anxiety about being perceived as aloof was measured by asking “How much anxiety do you experience about being perceived as aloof when you are not wearing a mask?” Responses were rated on a seven-point Likert scale (1 = not at all; 7 = very strongly).

We assumed that, among the younger generations in Japan, masks are used to hide one’s appearance for the purpose of security; some mask wearers in Japan have difficulty communicating with others face to face (Chiyoma, 2019). Anxiety about how self-appearance is perceived when not wearing a mask was measured by asking “How anxious do you feel about how your appearance is perceived by others when you are not wearing a mask?” Responses were rated on a seven-point Likert scale (1 = not at all; 7 = very strongly).

Sense of coherence (SOC) was measured by adapting the SOC-3UTHS scale, which was developed by Togari et al. (2007). The SOC contains three domains: comprehensibility, meaningfulness, and manageability. These domains were conceptualized as salutogenic factors by Antonovsky (1993). SOC was measured by asking “I believe I can find solutions to everyday problems and challenges,” “I believe some of the challenges and problems in life are worth facing and working through” and “I think I can understand and anticipate everyday problems and challenges.” Responses were rated on a seven-point Likert scale (1 = not at all; 7 = very strongly).

Studies conducted during the COVID-19 pandemic reported that SOC was associated with health-related outcomes, such as psychological well-being (Kayi et al., 2023), fewer health complaints (Dadaczynski et al., 2022), and lower anxiety (Leung et al., 2022). Therefore, in this study, SOC was considered a potential indicator of mask-wearing behavior.

Health literacy (HL) is associated with self-related risk perception (Wismans et al., 2022), which is manifested in mask-wearing behaviors (Zhang et al., 2022), vaccine intention (Latkin et al., 2021), and fewer misinformation beliefs about COVID-19 vaccination (McCaffery et al., 2020). In this study, HL was tested to confirm its association with mask-wearing behavior in the post-mask-mandate period by using the Communicative and Critical Health Literacy Scale, developed by Ishikawa et al. (2008). HL consists of five questions, including scale measures a person’s ability to effectively gather, evaluate, and use information for health-related decision-making. It includes the ability to: (1) collect information from various sources such as newspapers, books, TV, and the internet; (2) identify relevant information from a large amount of data; (3) assess the reliability of information; (4) understand and communicate information; and (5) make plans or take actions to improve health based on the information. Responses were rated on a five-point Likert scale (1 = not at all; 7 = very strongly).

### 2.4 Online survey procedure

The online questionnaire was distributed between June 12 and 24, 2023, 5-to-6 weeks after the relaxation of the mask-wearing regulations announced by the government of Japan. The survey was conducted more than 18 days after the announcement of the mask mandate relaxation, as habitual behavior tends to settle between 18 and 254 days (Lally et al., 2010). In addition, the survey was conducted before the rainy season began, as high humidity may have led people to intentionally remove their masks. Conducting a survey during this period may provide valuable insights for determining the optimal timing for governmental public health advisories aimed at discouraging excessive mask use.

### 2.5 Statistical analysis

Statistical analyses were performed using SPSS Statistics Version 25 (IBM Corp., Armonk, NY, USA). The Wilcoxon rank-sum test, Spearman’s rank correlation coefficient, and Pearson correlation coefficient were used for bivariate analyses. A multiple regression (Ordinary Least Squares regression) analysis was used to develop a model to indicate the frequencies and habits of mask wearing among respondents. In the multiple regression analysis, variables indicating a significant association between mask-wearing frequency and SHRI scores were chosen as independent variables. The variation inflation factor (VIF) scale was used to identify multicollinearity trends.

### 2.6 Ethical considerations

Ethics approval was obtained from the Biomedical Sciences Ethics Board of Nagasaki University (permission number: 23120701). The participants provided informed consent by clicking a button stating “I agree to participate in this study” prior to answering the online questionnaire; otherwise, they could not access the survey forms. The use of digital devices for obtaining informed consent was approved by the Ethical Guidelines for Life Sciences and Medical Research Involving Human Subjects authorized by the Ministry of Education, Culture, Sports, Science and Technology (MEXT), the Ministry of Health, Labor and Welfare (MHLW), and the Ministry of Economy, Trade and Industry (METI) of Japan.

## 3 Results

A total of 489 students participated in this study. Twelve fifth- and sixth-year students in medical and dental courses were excluded because their curriculum differed from that of four-year-college students. The fifth- and sixth-year students in medical and dental programs are engaged in long-term clinical practicums, during which they are required to wear masks daily. Therefore, 471 students were included in the analysis. The valid response rate was 23.6%. The respondents’ characteristics are listed in Table 1. Among the respondents, 44% were accustomed to wearing masks before the COVID-19 pandemic due to medical conditions such as allergies to pollen or rhinitis. Nearly 30% of the respondents answered that they needed to wear masks when participating in practicums or working part-time, whereas only 5.7% responded that they needed to wear masks due to medical conditions, such as allergies to pollen, at the time of the survey.

**Table 1:** Characteristics of the respondents (*N* = 471).

		*n*	%
Gender	Men	217	(46.1)
	Women	247	(52.4)
	NA	7	(1.5)
Grade	First	155	(32.9)
	Second	106	(22.5)
	Third	106	(22.5)
	Fourth	104	(22.1)
Medical/health sciences course	Yes	134	(28.5)
	No	337	(71.5)
Need to wear masks when required (i.e., practicum or part-time job)	Yes	140	(29.7)
	No	318	(67.5)
	NA	13	(2.8)
Used to wearing masks before the COVID-19 pandemic	Yes	205	(43.5)
	No	253	(53.7)
	NA	13	(2.8)
Need to wear masks due to medical conditions (i.e., rhinitis)	Yes	27	(5.7)
	No	431	(91.5)
	NA	13	(2.8)

**Figure 1:**
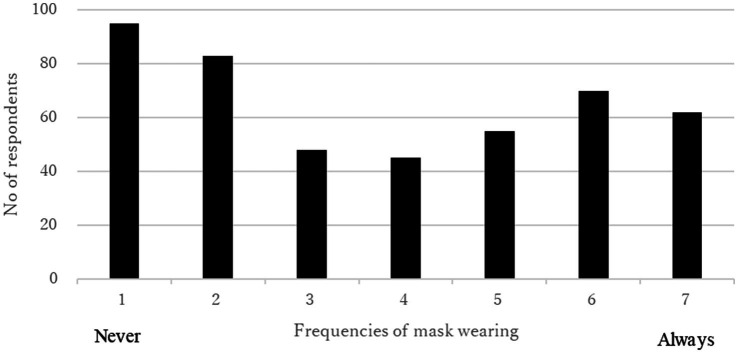
Mask wearing behaviors of the respondents.

**Figure 2:**
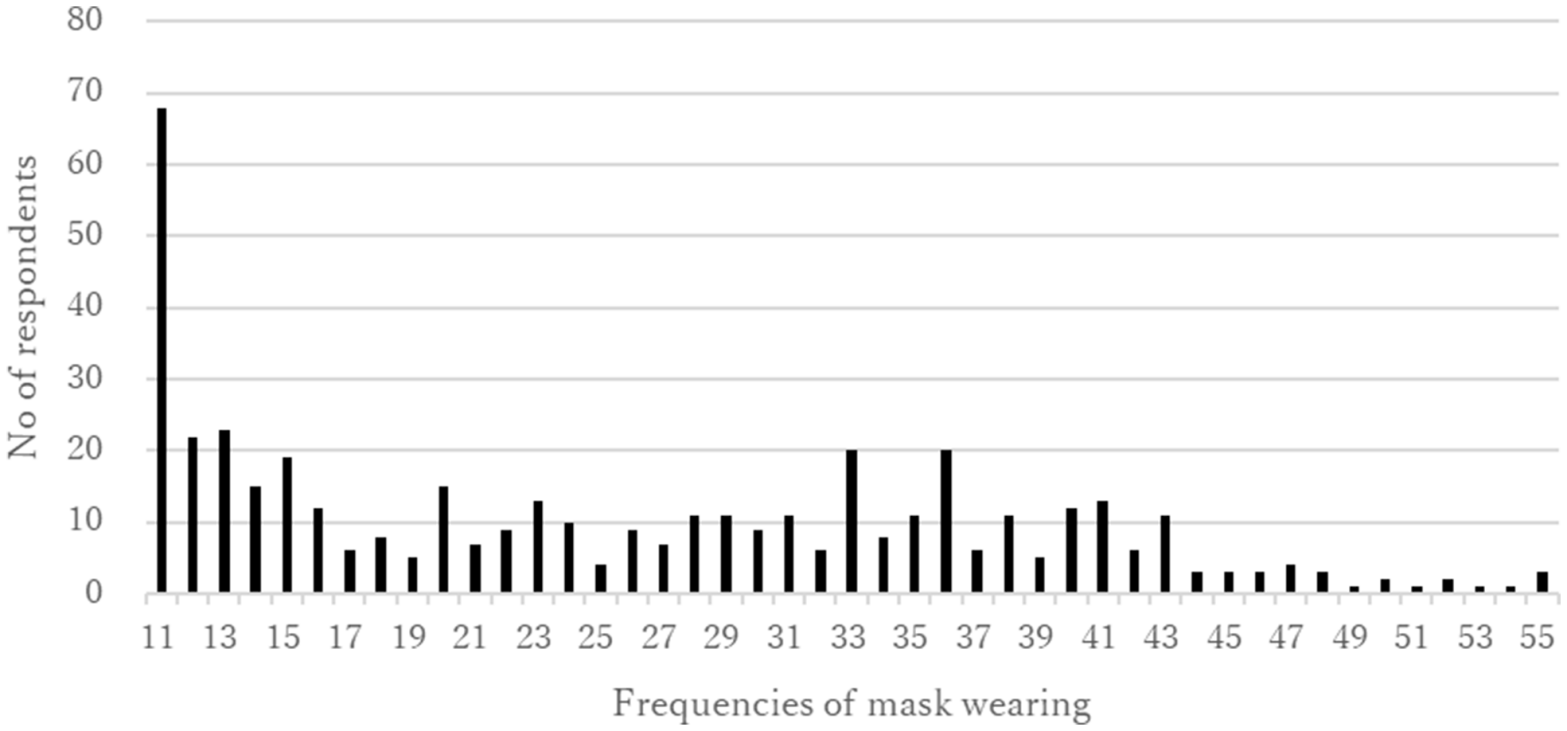
Mask wearing SRHI score.

The average score of the frequencies of wearing masks when going out was 3.74 (standard deviation [SD] 2.14), and the average mask-wearing SRHI score was 25.33 (SD 11.90). The frequency of wearing masks exhibited a dichotomous trend (Figure 1). The frequency of SHRI scores was concentrated in the lowest category (scores of 11–15), accounting for 32.7% of all respondents (Figure 2).

In terms of the type of anxiety experienced by respondents, anxiety about how their appearance was perceived when not wearing masks had the highest score of 3.41 (SD 1.97), followed by a sense of uneasiness when not wearing masks (3.34, SD 1.90), anxiety about being perceived as aloof when not wearing masks (3.00, SD 1.75), and anxiety about being infected by COVID-19 when not wearing masks (2.96, SD 1.68; Table 2).

**Table 2:** Anxiety scores of the respondents.

	Average	SD
Anxiety about how my appearance is perceived when not wearing masks	3.41	(1.97)
Sense of uneasiness when not wearing masks	3.34	(1.90)
Anxiety about being perceived as aloof when not wearing masks	3.00	(1.75)
Anxiety about being infected by COVID-19 when not wearing masks	2.96	(1.68)

SD, Standard Deviation.

The average SOC and HL scores were 14.86 (SD 2.97) and 18.14 (SD 3.55), respectively.

The Wilcoxon rank-sum test indicated that women ranked higher in both mask-wearing frequency (*p* < 0.001) and SRHI (*p* < 0.001) scores. Medical students scored higher on both mask-wearing frequency (*p* = 0.009) and SHRI (*p* = 0.017). Those who had medical conditions that required mask wearing had a higher rank in mask-wearing frequency (*p* = 0.038), whereas this tendency was not observed with the SRHI score. Respondents who were required to wear masks when participating in practicums or working part-time jobs had higher SHRI scores than their counterparts (*p* = 0.025). In addition, respondents who wore masks before the COVID-19 pandemic had higher SHRI scores than those who did not wear masks (*p* = 0.002; Table 3).

**Table 3:** Mask-wearing behaviors by characteristics of the respondents.

		Frequencies of mask wearing		Mask wearing SRHI score	
		Mean rank	*p* value	Mean rank	*p* value
Gender	Men	200.71	*p* < 0.001	193.68	*p* < 0.001
	Women	247.45		245.31	
Medical/health sciences course	Yes	255.18	*p* = 0.009	249.06	*p* = 0.017
	No	219.54		216.44	
Need to wear masks when required (i.e., practicum or part-time job)	Yes	218.96	*p* = 0.252	204.99	*p* = 0.025
	No	234.14		234.67	
Used to wearing masks before the COVID-19 pandemic	Yes	242.67	*p* = 0.052	246.56	*p* = 0.002
	No	218.83		208.50	
Need to wear masks due to medical conditions (i.e., rhinitis)	Yes	280.15	*p* = 0.038	258.94	*p* = 0.176
	No	226.33		223.45	

Moreover, for each type of anxiety, we examined the associations between gender, academic courses, whether individuals were required to wear masks when participating in practicums or working part-time jobs, whether they had been wearing masks before the COVID-19 pandemic, and whether they had medical conditions that required mask wearing (Table 4). Women scored higher than men on each anxiety subscale (*p* < 0.001).

**Table 4:** Anxiety scores by characteristics of the respondents.

		Anxiety of been infected by COVID-19 when not wearing masks		Sense of uneasiness when not wearing masks		Anxiety of been perceived as loof when not wearing masks		Anxiety about how my appearance is perceived when not wearing masks	
		Mean rank	*p* value	Mean rank	*p* value	Mean rank	*p* value	Mean rank	*p* value
Gender	Male	200.60	*p* < 0.001	195.54	*p* < 0.001	189.06	*p* < 0.001	185.25	*p* < 0.001
	Female	247.55		251.84		257.34		260.57	
Medical/Health Sciences Course	Yes	246.41	*p* = 0.081	249.34	*p* = 0.042	263.54	*p* < 0.001	256.10	*p* = 0.006
	No	222.94		221.80		216.30		219.10	
Need to wear masks when in required (i.e., practicum, parttime job)	Yes	221.07	*p* = 0.355	217.01	*p* = 0.172	207.62	*p* = 0.016	212.50	*p* = 0.063
	No	233.21		235.00		239.13		236.98	
Get use to wearing masks even before COVID-19 pandemic	Yes	246.77	*p* = 0.010	251.10	*p* = 0.001	241.12	*p* = 0.084	242.67	*p* = 0.051
	No	215.51		212.00		220.09		218.83	
Need to wear masks due to medical conditions (i.e.rhinitis)	Yes	261.89	*p* = 0.180	270.33	*p* = 0.092	215.39	*p* = 0.560	213.67	*p* = 0.514
	No	227.47		226.94		230.38		230.49	

Table 5 shows the association between mask-wearing frequency, SHRI scores, anxiety types, HL, and SOC. The sense of uneasiness due to not wearing masks was strongly associated with mask-wearing frequencies (rs = 0.764, *p* < 0.001) and SHRI scores (rs = 0.808, *p* < 0.001). Neither the frequency of mask wearing nor SRHI scores were significantly associated with HL or SOC.

**Table 5:** Correlations between anxiety scores, HL, SOC, and mask-wearing behaviors.

	1	2	3	4	5	6	7
1. Anxiety of been infected by COVID-19 when not wearing masks							
2. Sense of uneasiness when not wearing masks	0.706**						
3. Anxiety of been perceived as loof when not wearing masks	0.515**	0.621**					
4. Anxiety about how my appearance is perceived when not wearing masks	0.418**	0.655**	0.629**				
5. HL	−0.023	−0.018	−0.039	−0.006			
6. SOC	−0.085	−0.063	−0.130**	−0.133**	0.301**		
7. Frequencies of mask wearing	0.607**	0.764**	0.472**	0.545**	−0.002	0.001	
8. Mask wearing SRHI score	0.612**	0.808**	0.543**	0.637**	−0.027	−0.075	0.839**

^**^p < 0.01.

A multiple regression analysis was conducted to develop a model indicating the frequencies and habits of mask wearing among the respondents. To avoid multicollinearity, anxiety about being perceived as aloof when not wearing masks was excluded from the analysis. To assess the appropriateness of including the variable ‘sense of uneasiness when not wearing masks’ in the model, the VIF was below 2.741, which is considered statistically acceptable. The strongest indicator of both frequencies and habits of mask wearing was sense of uneasiness due to not wearing masks (*β* = 0.594, *p* < 0.001 and *β* = 0.623, *p* < 0.001 respectively). This suggests that unease is a significant motivator for mask-wearing behavior. Surprisingly, COVID-19-related anxiety had weak associations with both the frequencies and habits of mask wearing (*β* = 0.155, *p* < 0.001 and *β* = 0.089, *p* = 0.023 respectively; Table 6).

**Table 6:** Factors associate with mask-wearing behaviors.

	Frequencies of mask wearing		Mask-wearing SRHI score	
	*β*	*p* value	*β*	*p* value
Sense of uneasiness when not wearing masks	0.594	*p* < 0.001	0.623	*p* < 0.001
Anxiety about being infected by COVID-19 when not wearing masks	0.155	*p* < 0.001	0.089	*p* = 0.023
Anxiety about how my appearance is perceived when not wearing masks	0.091	*p* = 0.024	0.185	*p* < 0.001
Adjusted *R*^2^	0.592	*p* < 0.001	0.658	*p* < 0.001
Control variables: gender, academic course				

## 4 Discussion

This study examined the mask-wearing frequency and habitual behaviors at the point of the relaxation of mask-wearing mandates by the government. The frequency of wearing masks exhibited a dichotomous trend, which indicated a clear division between those who continued to wear masks after the relaxation of mask-wearing mandates and those who did not. However, more than 30% of the respondents were unlikely to exhibit habitual mask-wearing behavior. This indicated that the respondents were unlikely to continue habitually wearing masks after the 3 years of mask-wearing mandates were relaxed. This could suggest that the respondents wished to stop wearing masks, despite government recommendations to wear masks to prevent viral exposure after regulations were relaxed. This was in line with the finding that anxiety about being infected with COVID-19 when not wearing masks had the lowest score among the four types of anxiety. Respondents with lower anxiety about infection were less likely to wear masks.

We found common sociodemographic characteristics associated with the frequency of wearing masks and SHRI scores. Women were more likely than men to wear masks and develop habitual mask-wearing behaviors. This may be attributed to health concerns and anxiety among women regarding appearance (Miyazaki et al., 2021) and viral exposure (Ayran et al., 2022; Vest et al., 2022; Zhang et al., 2022). Our study further suggests that some motivations differ by gender. Howard (2021) reported that men were more likely to perceive facemasks as infringing on their independence, whereas women were more likely to perceive facemasks as uncomfortable. In addition, our study indicated that women were more likely than men to wear masks and develop a habit of wearing them. Furthermore, women were more anxious about not wearing masks for various reasons beyond virus exposure, such as sense of uneasiness, fear of being perceived as aloof, and concern about their appearance. These results were in line with those presented by Alsharawy et al. (2021) and Galasso et al. (2020), who investigated eight countries and found that women are more likely to perceive the pandemic as a serious health problem and to agree and comply with restraining measures. Similarly, in this study, women were found to be likely to take every measure, including mask wearing, to protect themselves; thus, they were more likely to develop mask wearing as a habit. This phenomenon seems to have led to “mask addiction,” which was pointed out by Chiyoma (2019) based on the evidence that wearing masks quite often even without health-related concerns, among young women in Japan.

Moreover, the academic courses that respondents were enrolled in were associated with mask wearing frequencies and SRHI scores. Students in medical and health sciences courses wore masks more frequently and were more likely to develop mask-wearing habits than students in other courses. These findings suggest that students in medical and health sciences courses were more aware of the threat of COVID-19 because they were well-educated about its potential harm and damage to the body.

Unlike the respondents’ gender and academic courses, the urgent need to wear masks seemed to be differently associated with the frequency of mask wearing and mask-wearing habits. For instance, medical conditions at the time of the survey, such as seasonal rhinitis, significantly affected the frequency of wearing masks but did not significantly impact the SRHI scores. Wearing masks to prevent pollen exposure, which leads to allergic reactions, is common in Japan, particularly during spring (Chiyoma, 2019; Kamatani et al., 2021). Masks are effective in preventing the transmission of pollen (Wagner et al., 2022). People in Japan who wear masks have been found to be 60% less likely to be infected by COVID-19 than those who do not (Sugimura et al., 2021). However, mask-wearing behavior for acute medical conditions is temporary and may be less likely to become a habit; people who wear masks to cope with acute medical syndromes seem to stop wearing masks once these conditions change.

However, mask-wearing habits may have been influenced by long-term habits established before this study. This was evidenced by the fact that respondents who were accustomed to wearing masks before the COVID-19 pandemic were likely to have higher SRHI scores than those who were not. Interestingly, respondents who were required to wear masks during practicums or part-time jobs had lower SRHI scores than those who were not. The authors’ interpretation of this fact is as follows: The individuals who are required to wear masks because of circumstances such as practicum sessions or part-time jobs are more likely to wear masks consciously. In such situations, mask-wearing is seen as being mandatory, and they are aware that they must follow the rules. Thus, their mask-wearing behavior is driven by deliberate action rather than by unconscious habit. On the other hand, individuals who are not required to wear masks tend to do so unconsciously. As a result, the SRHI scores of those not required to wear masks were higher than those of individuals who were required to wear them.

One of the contributions of this study is the finding that anxieties leading to wearing masks influenced both the frequency of mask wearing and SRHI scores for mask wearing, even after controlling for gender and academic courses. Furthermore, the results revealed differences in the structure between mask-wearing behavior and SRHI scores for mask wearing in terms of the strength of the effects of each independent variable on each dependent variable. The frequency of mask wearing was strongly influenced by the sense of uneasiness when not wearing masks and, to a lesser extent, by anxiety about being infected with COVID-19 as well as concerns about how one’s appearance is perceived without a mask. In contrast, for SRHI scores, the sense of uneasiness was the strongest indicator, followed by concerns about how one’s appearance was perceived without a mask and a weaker influence of anxiety about being infected with COVID-19. The sense of uneasiness, which was identified as the strongest indicator of both the frequency and SRHI score of mask wearing, represented a feeling of uncertainty and anxiety. The vague sense of uneasiness felt when not wearing masks can be understood as a cognitive process that includes an indistinct awareness, as highlighted by Miyake (2007). In other words, anxiety involves a cognitive process that includes indistinct awareness, unlike other types of anxiety with clear targets, such as fear of contracting COVID-19 or concern about how one’s appearance is evaluated by others. This type of vague anxiety can cause individuals to be unsure of the reason for their anxiety, making it difficult for them to adopt appropriate coping strategies. Consequently, people might wear masks as a provisional coping behavior to achieve a sense of security, leading them to feel somewhat reassured by wearing masks.

In addition, the anxiety about being infected with COVID-19 when not wearing masks, which marked the lowest average score compared with other types of anxieties, suggested that the respondents of this study felt less anxious about contracting the virus than about how their appearance was perceived or being seen as aloof when not wearing masks. The survey was conducted when the government of Japan warned of a rising ninth wave of COVID-19 through the mass media due to an increase in the number of people who had stopped wearing masks. Therefore, the respondents in this study appeared to wear masks only provisionally. However, this provisional behavior contributed less to the development of habitual mask-wearing practices for several reasons. Habits are fixed behaviors acquired through repetition that can be performed with minimal mental effort. Anxiety about being infected with COVID-19 appears to be influenced by pandemic conditions that are constantly changing. Therefore, habitual mask-wearing behavior is less likely to be affected, particularly after the official declaration of mask-wearing relaxation.

Anxiety about how one’s appearance is perceived when not wearing a mask was likely influenced by subjective perceptions of the self, such as self-efficacy (Bandura, 1994), rather than pandemic conditions, which were objective. Thus, it was less influenced by the frequency of mask wearing than anxiety about being infected by COVID-19 at the time of the survey, which was conducted when the ninth wave was arising. However, this type of anxiety is subjective and based on an individual’s fixed perception. Therefore, those experiencing such anxiety seem to cope by wearing masks and habitualizing the behavior of wearing masks to conceal their appearance.

The present study identified indicators of mask-wearing behaviors and variables that did not contribute as indicators of such behaviors. We found that HL and SOC were not associated with the frequency of mask wearing or mask-wearing SRHI scores. Furthermore, HL and SOC did not influence mask-wearing behavior. This suggested that the motivation for wearing masks in this context was influenced more by psychological comfort and social norms than by knowledge or comprehension of health information. In other words, wearing masks may not have been a behavior to protect oneself from invisible viruses that risk one’s health. Respondents wore masks to protect themselves from the perceived invisible discomfort or anxiety they experienced in society. This behavior is consistent with mask usage to seek psychological comfort by concealing the lower parts of the face to hide mask-wearers’ emotions in nonverbal communication, as noted by Chiyoma (2019).

However, wearing masks retains moisture; therefore, one must be cautious about the risk of heatstroke when wearing face masks, especially during hot and humid summers. Changing people’s behavior, such as getting them to stop wearing masks, can be difficult. Therefore, the government should develop a heatstroke prevention proposal similar to that created for medical personnel (Working Group on Heatstroke Medical Care during the COVID-19 Epidemic, 2020) to warn the public about the risk of heatstroke when wearing masks.

This study had several limitations. First, the results may have been affected by sampling bias. The response rate was 23.6%, and the target population was limited to university students; therefore, one must be cautious when applying the model of mask-wearing behavior to the general population, especially to those in professions that require mandatory mask use, such as healthcare workers. Also, caution must be exercised when applying this model, which is based on the younger generation such as university students, to older individuals—the majority of Japan’s population—who tend to exhibit more pronounced physical and mental symptoms that may influence their mask-wearing behavior. Second, this study was conducted 5–6 weeks after the relaxation of the mask-wearing mandate; however, it may not fully capture long-term behavioral changes. Further research is needed to identify the timing of interventions that may effectively promote sustained changes in mask-wearing behavior. Third, to avoid multicollinearity, anxiety about being perceived as aloof when not wearing a mask was excluded from the model. Therefore, caution must be exercised when interpreting this model. Further studies are needed to test models of mask-wearing behavior by including factors related to anxiety about being perceived as aloof when not wearing masks in the general population.

## 5 Conclusion

This study examined the factors influencing the continued habitual behaviors and frequency of mask wearing in Japan after the relaxation of COVID-19 mask mandates. The results suggest that addressing psychological comfort and social norms may be more effective in promoting mask use than focusing on disease-related anxiety. Considering the recurring nature of viral outbreaks, healthy lifestyles that consider diverse perceptions of mask use must be established. Further research is required to demonstrate the applicability of our findings beyond university students to the general population and to countries outside Japan, where cultural and other variables may differentially influence mask wearing.

## Data Availability

The raw data supporting the conclusions of this article will be made available by the authors, without undue reservation.

## References

[ref1] Alsharawy A. Spoon R. Smith A. Ball S. (2021). Gender differences in fear and risk perception during the COVID-19 pandemic. Front. Psychol. 12:689467. doi: 10.3389/fpsyg.2021.689467, PMID: 34421741 PMC8375576

[ref2] Antonovsky A. (1993). The structure and properties of the sense of coherence scale. Soc. Sci. Med. 36, 725–733. doi: 10.1016/0277-9536(93)90033-z, PMID: 8480217

[ref3] Ayran G. Köse S. Sarıalioğlu A. Çelebioğlu A. (2022). Hand hygiene and mask-wearing behaviors and the related factors during the COVID 19 pandemic: a cross-sectional study with secondary school students in Turkey. J. Pediatr. Nurs. 62, 98–105. doi: 10.1016/j.pedn.2021.10.001, PMID: 34688528 PMC8491930

[ref4] Bandura A. (1994). “Self-efficacy” in Encyclopedia of human behavior. ed. Ramachandran V. S., vol. 4 (New York: Academic Press), 71–81.

[ref5] Cha S. E. Ku X. Choi I. (2023). Post COVID19, still wear a face mask? Self-perceived facial attractiveness reduces mask-wearing intention. Front. Psychol. 14:1084941. doi: 10.3389/fpsyg.2023.1084941, PMID: 36760455 PMC9904203

[ref6] Chiyoma I. (2019). Masks in Japan: an investigation of background and roles. Paper Lang. Literature Cult. 19, 81–91.

[ref7] Crimon C. Barbir M. Hagihara H. de Araujo E. Nozawa S. Shinya Y.. (2022). Mask wearing in Japanese and French nursery schools: the perceived impact of masks on communication. Front. Psychol. 13:874264. doi: 10.3389/fpsyg.2022.874264, PMID: 36420380 PMC9677818

[ref8] Dadaczynski K. Okan O. Messer M. Rathmann K. (2022). University students’ sense of coherence, future worries and mental health: findings from the German COVID-HL-survey. Health Promot. Int. 37:daab070. doi: 10.1093/heapro/daab070, PMID: 34214156 PMC8851400

[ref9] Ekmektzoglou K. Tziatzios G. Siau K. Pawlak K. M. Rokkas T. Triantafyllou K.. (2021). Covid-19: exploring the "new normal" in gastroenterology training. Acta Gastroenterol. Belg. 84, 627–635. doi: 10.51821/84.4.014, PMID: 34965045

[ref10] Freud E. Di Giammarino D. Camilleri C. (2022). Mask-wearing selectivity alters observers’ face perception. Cogn. Res. Princ. Implic. 7:97. doi: 10.1186/s41235-022-00444-z, PMID: 36380225 PMC9666572

[ref11] Galasso V. Pons V. Profeta P. Becher M. Brouard S. Foucault M. (2020). Gender differences in COVID-19 attitudes and behavior: panel evidence from eight countries. Proc. Natl. Acad. Sci. USA 117, 27285–27291. doi: 10.1073/pnas.2012520117, PMID: 33060298 PMC7959517

[ref12] Goldberg M. H. Gustafson A. Maibach E. W. Ballew M. T. Bergquist P. Kotcher J. E.. (2020). Mask-wearing increased after a government recommendation: a natural experiment in the U.S. during the COVID-19 pandemic. Front. Commun. 5:44. doi: 10.3389/fcomm.2020.00044

[ref13] Horii M. Why do the Japanese wear masks? A short historical review. Electron. J. Contemp. Jpn. Stud.. (2014), 14. Available online at: https://www.japanesestudies.org.uk/ejcjs/vol14/iss2/horii.html

[ref14] Howard M. C. (2021). Gender, face mask perceptions, and face mask wearing: are men being dangerous during the COVID-19 pandemic? Pers. Individ. Dif. 170:110417. doi: 10.1016/j.paid.2020.110417, PMID: 33052155 PMC7543707

[ref15] Ishikawa H. Nomura K. Sato M. Yano E. (2008). Developing a measure of communicative and critical health literacy: a pilot study of Japanese office workers. Health Promot. Int. 23, 269–274. doi: 10.1093/heapro/dan017, PMID: 18515303

[ref16] Kamatani M. Ito M. Miyazaki Y. Kawahara J. I. (2021). Effects of masks worn to protect against COVID-19 on the perception of facial attractiveness. i-Perception 12:20416695211027920. doi: 10.1177/20416695211027920, PMID: 34262683 PMC8243111

[ref17] Kayi I. Uzunköprü G. Dadaczynski K. Soylar P. Otludil B. Dündar P.. (2023). Gender differences in sense of coherence among university students during the COVID-19 pandemic in Turkey. Health Promot. Int. 38:daad048. doi: 10.1093/heapro/daad048, PMID: 37279469 PMC10243759

[ref18] Lally P. Jaarsved C. H. M. V. Potts H. W. W. Wardle J. (2010). How are habits formed: modelling habit formation in the real world. Eur. J. Soc. Psychol. 40, 998–1009. doi: 10.1002/ejsp.674

[ref19] Latkin C. A. Dayton L. Yi G. Colon B. Kong X. (2021). Mask usage, social distancing, racial, and gender correlates of COVID-19 vaccine intentions among adults in the US. PLoS One 16:e0246970. doi: 10.1371/journal.pone.0246970, PMID: 33592035 PMC7886161

[ref20] Leung A. Y. M. Parial L. L. Tolabing M. C. Sim T. Mo P. Okan O.. (2022). Sense of coherence mediates the relationship between digital HL and anxiety about the future in aging population during the COVID-19 pandemic: a path analysis. Aging Ment. Health 26, 544–553. doi: 10.1080/13607863.2020.1870206 33438448

[ref21] Lewis J. Mc Auliffe S. O’Sullivan K. O’Sullivan P. Whiteley R. (2021). Musculoskeletal physical therapy after COVID-19: time for a new "normal". J. Orthop. Sports Phys. Ther. 51, 5–7. doi: 10.2519/jospt.2021.0102 33383997

[ref22] Mack H. G. Fraser-Bell S. (2021). COVID new normal in ophthalmology: implications for ophthalmologists, eye care, ophthalmic education and research. Clin. Experiment. Ophthalmol. 49, 9–11. doi: 10.1111/ceo.13898, PMID: 33462979 PMC8013672

[ref23] Manuti A. Van der Heijden B. Kruyen P. De Vos A. Zaharie M. Lo Presti A. (2022). Editorial: how normal is the new normal? Individual and organizational implications of the COVID-19 pandemic. Front. Psychol. 13:931236. doi: 10.3389/fpsyg.2022.931236, PMID: 35800919 PMC9253824

[ref24] Maru V. (2021). The “new normal” in post-COVID-19 pediatric dental practice. Int. J. Paediatr. Dent. 31, 528–538. doi: 10.1111/ipd.12764, PMID: 34148269 PMC8447441

[ref25] Mashiko H. (2010). Influence of external over-adaptive Behavior and introspection on sense of authenticity among university student. J. Sch. Ment. Heal. l3, 19–26. doi: 10.24503/jasmh.13.1_19

[ref26] McCaffery K. J. Dodd R. H. Cvejic E. Ayrek J. Batcup C. Isautier J. M.. (2020). Health literacy and disparities in COVID-19-related knowledge, attitudes, beliefs and behaviours in Australia. Public Health Res. Pract. 30:30342012. doi: 10.17061/phrp30342012, PMID: 33294907

[ref27] Ministry of Health, Labor and Welfare. (2023). Response to COVID-19 after the classification change. Available online at: https://www.mhlw.go.jp/stf/corona5rui.html (Accessed August 25, 2024).

[ref28] Miyake S. (2007). Some aspects of the meaning of anxiety: focusing on the significance of anxiety measured using the self-reported questionniare technique. Jpn. Psychol. Rev. 50, 402–419.

[ref29] Miyazaki Y. Kamatani M. Kawahara J. (2021). The influence of social anxiety, trait anxiety, and perceived vulnerability to disease on the frequency of face mask wearing. Jpn. J. Psychol. 92, 339–349. doi: 10.4992/jjpsy.92.20063

[ref30] Nakayachi K. Ozaki T. Shibata Y. Yokoi R. (2020). Why do Japanese people use masks against COVID-19, even though masks are unlikely to offer protection from infection? Front. Psychol. 11:1918. doi: 10.3389/fpsyg.2020.01918, PMID: 32849127 PMC7417658

[ref31] Ribeiro V. V. Dassie-Leite A. P. Pereira E. C. Santos A. D. N. Martins P. Irineu R. A. (2022). Effect of wearing a face mask on vocal self-perception during a pandemic. J. Voice 36, 878.e1–878.e7. doi: 10.1016/j.jvoice.2020.09.006, PMID: 33011037 PMC7527314

[ref32] Saito J. (2020). The “new normal” in the COVID-19 era: Temporary or permanent? Available online at: https://www.jcer.or.jp/english/the-new-normal-in-the-covid-19-era-temporary-or-permanent (Accessed August 25, 2024).

[ref33] Sakakibara R. Ozono H. (2021). Why do people wear a mask? A replication of previous studies and examination of two research questions in a Japanese sample. Jpn. J. Psychol. 92, 332–338. doi: 10.4992/jjpsy.92.20323

[ref34] Sugimura M. Chimed-Ochir O. Yumiya Y. Ohge H. Shime N. Sakaguchi T.. (2021). The association between wearing a mask and COVID-19. Int. J. Environ. Res. Public Health 18:9131. doi: 10.3390/ijerph18179131, PMID: 34501719 PMC8431493

[ref35] Takami K. (2023). Development of Japanese version of the self-report habit index. Bull. Grad. Sch. Hum. Dev. Environ. Kobe Univ. 16, 29–39. doi: 10.24546/0100481767

[ref36] Togari T. Yamazaki Y. Nakayama K. Shimizu J. (2007). Development of a short version of the sense of coherence scale for population survey. J. Epidemiol. Community Health 61, 921–922. doi: 10.1136/jech.2006.056697, PMID: 17873231 PMC2652976

[ref37] Ueno S. (2021). Physiological burden by wearing a mask. Jpn. J. Occup. Med. Traumatol. 69, 1–8.

[ref38] Verplanken B. O. S. (2006). Reflections on past behavior: a self-report index of habit strength. J. Appl. Soc. Psychol. 33, 1313–1330. doi: 10.1111/j.1559-1816.2003.tb01951.x

[ref39] Vest J. R. Cash-Goldwasser S. Peters Bergquist E. Embi P. J. Caine V. Halverson P. K. (2022). Indoor public mask-wearing behavior changes in response to national, state, and local COVID-19 policies. J. Public Health Manag. Pract. 28, 292–298. doi: 10.1097/phh.0000000000001467, PMID: 34939598 PMC8963438

[ref40] Wagner J. Macher J. M. Chen W. Kumagai K. (2022). Comparative mask protection against inhaling wildfire smoke, allergenic bioaerosols, and infectious particles. Int. J. Environ. Res. Public Health 19:15555. doi: 10.3390/ijerph192315555, PMID: 36497628 PMC9735667

[ref41] Wang X. Chen H. Liu L. Liu Y. Zhang N. Sun Z.. (2020). Anxiety and sleep problems of college students during the outbreak of COVID-19. Front. Psych. 11:588693. doi: 10.3389/fpsyt.2020.588693, PMID: 33329134 PMC7719633

[ref42] World Health Organization. (n.d.). Coronavirus disease (COVID-19) advice for the public: When and how to use masks. Available online at: https://www.who.int/emergencies/diseases/novel-coronavirus-2019/advice-for-public/when-and-how-to-use-masks. [Accessed August 25, 2024].

[ref43] Wismans A. van der Zwan P. Wennberg K. Franken I. Mukerjee J. Baptista R.. (2022). Face mask use during the COVID-19 pandemic: how risk perception, experience with COVID-19, and attitude towards government interact with country-wide policy stringency. BMC Public Health 22:1622. doi: 10.1186/s12889-022-13632-9, PMID: 36028876 PMC9412789

[ref44] Working Group on Heatstroke Medical Care during the COVID-19 Epidemic (2020). Heatstroke management during the COVID‐19 epidemic: recommendations from the experts in Japan. Acute med. Surg. 7:e560. doi: 10.1002/ams2.560 32837733 PMC7436206

[ref45] Yoshikawa S. (2017). “Date-masuku (fake mask)” through psychological perspective (in Japanese). Bull. Hannan Univ. Humanit. Nat. Sci. 53, 35–40.

[ref46] Zhang W. Chen S. F. Li K. K. Liu H. Shen H. C. Zhang X. C. (2022). Mask-wearing behavior during COVID-19 in China and its correlation with e-health literacy. Front. Public Health 10:930653. doi: 10.3389/fpubh.2022.930653, PMID: 35937248 PMC9354616

